# Gut microbiota: key facilitator in metastasis of colorectal cancer

**DOI:** 10.3389/fonc.2023.1270991

**Published:** 2023-10-30

**Authors:** Siyi Yu, Shuyi Wang, Bin Xiong, Chunwei Peng

**Affiliations:** ^1^ Department of Gastrointestinal Surgery, Zhongnan Hospital of Wuhan University, Wuhan, China; ^2^ Hubei Key Laboratory of Tumor Biological Behaviors, Wuhan, China; ^3^ Hubei Cancer Clinical Study Center, Wuhan, China

**Keywords:** gut microbiota, colorectal cancer, metastasis, tumor progression, immune evasion

## Abstract

Colorectal cancer (CRC) ranks third in terms of incidence among all kinds of cancer. The main cause of death is metastasis. Recent studies have shown that the gut microbiota could facilitate cancer metastasis by promoting cancer cells proliferation, invasion, dissemination, and survival. Multiple mechanisms have been implicated, such as RNA-mediated targeting effects, activation of tumor signaling cascades, secretion of microbiota-derived functional substances, regulation of mRNA methylation, facilitated immune evasion, increased intravasation of cancer cells, and remodeling of tumor microenvironment (TME). The understanding of CRC metastasis was further deepened by the mechanisms mentioned above. In this review, the mechanisms by which the gut microbiota participates in the process of CRC metastasis were reviewed as followed based on recent studies.

## 1 Introduction

Colorectal cancer (CRC) has the third highest incidence rate among all types of cancers globally (1). The main cause of death of CRC patients is metastasis, which is also a clinical challenge (2, 3). Metastasis is a multi-step and multi-factor process including the separation of tumor cells from each other, invasion into surrounding tissues, adhesion to endothelial cells, and migration from the primary site to secondary site. Several mechanisms have been implicated, such as epithelial-mesenchymal transition (EMT) (4, 5), changes in expression of intercellular adhesion molecules (6), loss of structural integrity of the basement membrane (7), remodeling of the pre-metastatic niche (8), and induction of angiogenesis (9). Nonetheless, it is worth noting that current understanding of CRC couldn’t fully illuminate the role of systematic factors like exercise, diet and aging in CRC metastasis.

Gut microbiota located within the intestinal tract comprises a large and diverse community including bacteria, yeasts, fungi viruses and parasites, which are referred to as the second gene pool of the human body (10). As one of the earliest encountered foreign antigens in the human body, gut microbiota plays essential roles in various physiological and pathological processes. Previously, the main roles attributed to gut microbiota were the synthesis of essential amino acids and vitamins, the digestion of polysaccharides that are difficult to assimilate, and contribution to human metabolic processes (11). Additionally, gut microbiota provides essential signals for the development and functioning of immune system (12). In recent years, numerous studies have suggested that the gut microbiota also participates in oncogenesis and progression of cancer, particularly in the process of metastasis (13–15). On the one hand, the gut microbiota secretes various metabolites or virulence factors that damage host DNA (16) and contributes to a pro-inflammatory environment (17), leading to pre-cancerous lesions. On the other hand, the gut microbiota directly interacts with cancer cells, thereby increasing invasion and proliferation of cancer cells (18). Furthermore, several studies have indicated that the gut microbiota may facilitate metastasis by affecting the recruitment of immune cells and remodeling the tumor microenvironment (TME) (19–23). The mechanisms by which the gut microbiota participates in the process of CRC metastasis were reviewed as followed based on recent studies.

## 2 Gut microbiota promotes the proliferation and invasion of CRC cells

### 2.1 Gut microbiota promotes the proliferation of CRC cells

The progression of CRC involves multiple signaling pathways (24, 25). The disruption of cell cycle and the acquisition of unlimited proliferative capacity are key steps in cancer progression. Researches have indicated that Fusobacterium, a specific type of bacteria, has a significantly higher relative abundance in CRC tissue compared to normal one (26–28). The quantity of Fusobacterium also exhibits statistical differences between different stages of cancer progression. Furthermore, during the transition from adenoma to malignant tumor, the abundance of Fusobacterium gradually increases (29). Recent studies have demonstrated that the gut microbiota may promote cancer cell proliferation through mechanisms as follows.

#### 2.1.1 Modulating RNA-mediated targeting effects

RNA-mediated targeting effects are important mechanisms of epigenetic regulation (30), including the synthesis of various non-coding RNAs and their impact on downstream genes (31, 32). MicroRNAs (miRNAs) are one of the key players in this process, regulating various biological processes such as tumorigenesis. Recent studies have indicated that the gut microbiota is involved in RNA-mediated targeting effects that regulate cancer cell proliferation.

Fusobacterium not only facilitated the proliferation and invasiveness of co-cultured CRC cell lines but also promoted tumor formation in APCMin/+ mice. Fusobacterium activated the TLR4/MYD88 receptors on the surface of cancer cells, leading to the activation of NFκB. NFκB then binds to the upstream region of the transcription start site (TSS) of miR21, upregulating its expression. MiR21, in turn, bound to the 3’ end binding site of RASA1, inhibiting its expression (33). RASA1 is a member of the RAS GTPase-activating protein (RAS-GAP) family, and its binding to the well-known oncogenic protein RAS can inhibit RAS activity (34). Some studies have suggested that mutations or loss of function in RASA1 in CRC leads to activation of the RAS-MAPK cascade (35–37). The MAPK pathway is reported to induce the synthesis of cycling D1, promoting cell division (38). The MAPK pathway has also been shown to participate in the proliferation of cancer cells in multiple studies (39, 40).

Peptostreptococcus micros (P. micros) is an opportunistic pathogen found in the oral cavity that is closely associated with periodontitis (41). It can also cause suppurative infections in various organs throughout the body (41). Chang et al. found that P. micros could significantly foster the proliferation of LoVo and HT-29 cell lines *in vitro* (42). To unveil the underlying mechanism, they constructed xenograft models. It came out that tumors derived from cancer cells co-cultured with P. micros had larger volume and weight (42). Further investigations revealed that P. micros suppressed the expression of protein tyrosine phosphatase receptor R (PTPRR) by upregulating miR-218-5p, ultimately activating the Ras/ERK/cFos signaling pathway (42). The Ras/ERK signaling pathway is part of MAPK pathway and also participates in the proliferation of CRC cells (43).

Significantly associated with inflammatory bowel disease (IBD) and CRC, Enterotoxigenic Bacteroides fragilis (ETBF) is a molecular subtype of Bacteroides fragilis (44, 45). ETBF could downregulate the expression of miR-149-3p in cancer cell lines and influences the selective splicing of the KAT2A gene through PHF5A. Ultimately, KAT2A directly binds to the promoter region of SOD2, activating the SOD2 gene (46). SOD2 has been shown to modulate energy metabolism and promote proliferation of CRC (47).

#### 2.1.2 Activating the cascades of cancer signaling

The Wnt/β-catenin signaling pathway plays a crucial role in physiological processes such as cell proliferation and differentiation, stem cell renewal, embryonic development, and tissue homeostasis (48). Dysregulation of this pathway is widely considered a key oncogenic signal and is of significant importance in the development of different kinds of cancers (49). Certain bacteria, such as Fusobacterium nucleatum (F. nucleatum), could facilitate cancer cell proliferation through the Wnt/β-catenin pathway (18, 50). For example, F. nucleatum produces a virulence factor called FadA (51), which binds to the E-cadherin domain EC5 on the surface of CRC cells. This interaction leads to the dephosphorylation of β-catenin, accumulation of β-catenin in the cytoplasm, and translocation of β-catenin to the cell nucleus. Subsequently, the expression of transcription factors lymphoid enhancer-binding factor (LEF)/T-cell factor (TCF), NFκB, and oncogenes such as Myc and Cyclin D1 is upregulated, promoting CRC cell proliferation (18). Additionally, FadA could promote the expression of chk2 through the E-cadherin/β-catenin pathway, leading to increased DNA damage and elevated proliferative capacity in CRC cells (50). Furthermore, some studies have reported that probiotics have the ability to inhibit cancer cell proliferation and promote apoptosis (52–55). qPCR and western blot results have shown that during this process, the gene expression and protein content of β-catenin in CRC decrease, suggesting that probiotics may inhibit CRC cell proliferation by regulating β-catenin-related pathways (52). Nonetheless, the underlying mechanisms of these effects are still need to be explored.

The PI3K-Akt pathway is widely activated in various tumors and is closely associated with tumor development (56–59). Gram-positive anaerobic bacteria, such as Peptostreptococcus anaerobius (P. anaerobius), present in the oral cavity and intestines (60), could bind to integrin α2β1 on the surface of CRC cells through its surface protein called putative cell wall binding repeat 2 (PCWBR2). This interaction activates the PI3K-Akt signaling pathway through Focal Adhesion Kinase 9 (FAK9), ultimately promoting cancer cell proliferation (61).

The MAPK-ERK pathway, a cell proliferation signaling pathway located on the cell surface and extended to the nucleus, plays a crucial role in cell proliferation (62). Activation of the MAPK-ERK pathway is increasingly implicated in the occurrence and progression of CRC (63). The oral pathogenic bacterium Porphyromonas gingivalis (P. gingivalis), once colonizing the colon, can selectively invade CRC cells and activate the MAPK-ERK pathway, thereby promoting tumor proliferation (64).

Not only individual bacterial species but also the overall balance of the gut microbiota is crucial in regulating cancer proliferation. Bai et al. have found that smoking induced gut microbiota dysbiosis altered gut metabolites and impaired gut barrier function, ultimately activating the oncogenic MAPK-ERK signaling and enhancing cancer cell proliferation (65). Portulaca oleracea, a medicinal plant and a member of the Portulacaceae family, is well-known for its resistance against micro­biota, inflammation, and cancer (66). Portulaca oleracea extract (POE) has been found to reduce tumor quantity and improve survival rate in carcinogen-induced mouse models through restoring the balance of gut microbiota. Further results have shown that POE upregulates the expression of TP53, inhibits the Wnt/β-catenin signaling pathway and reduces the expression of c-Myc and Cyclin D1, ultimately suppressing cancer cell proliferation (67).

### 2.2 The gut microbiota promotes the invasiveness of cancer cells

In addition to unlimited proliferative capacity, invasive growth into surrounding tissues is another characteristic of malignant tumors. Breaking through the basement membrane is the first step for distant metastasis (68). It has been shown that a positive correlation between the gut microbiota and tumor progression stages exists (29). Since one of the defining criteria for tumor progression stages is the depth of tumor infiltration (69), the gut microbiota has the potential to regulate the invasive properties of cancer cells.

#### 2.2.1 Secreting microbiota-derived functional substances

The metabolic products derived from microorganisms, such as l-2-hydroxyglutarate, succinate, fumarate, d-2-hydroxyglutarate, and lactate, can accumulate in tumor lesions and exacerbate the malignancy of the tumor (70). Furthermore, some metabolites could hijack signaling pathways related to tumor metastasis through gene regulation (71). Formate, a major metabolic product of F. nucleatum, can activate the AhR signaling pathway in CRC, enhancing its cancer stem cell properties and increasing the invasiveness of CRC, ultimately promoting cancer metastasis (72).

EMT is a cellular biological process (73, 74) that endows cancer cells with invasive and anti-apoptotic capabilities (75, 76). EMT triggers the process of dissemination and invasion, ultimately leading the formation of metastases (77, 78). Certain strains of Escherichia coli can produce a virulence protein called cytotoxic necrotizing factor 1 (CNF1) (79). CNF1 induces the recruitment of mTOR to lysosomes, consequently increasing invasiveness of CRC cell lines and inducing the expression of EMT markers (80). These findings suggest that gut microbiota has the potential to induce EMT in cancer cells.

Hydrogen sulfide (H_2_S) has been identified as the third gasotransmitter after nitric oxide (NO) and carbon monoxide (CO) and participates in a variety of biological processes (81). There are two sources of luminal H_2_S: the inorganic and organic metabolism of intestinal bacteria (82) and endogenously synthesized in the mammal cells (83). Endogenous H_2_S fosters metastasis, partly through induction of ATP citrate lyase (ACLY) to facilitate EMT (84, 85). Since the luminal H_2_S mainly originate from bacterial metabolism and directly contact with intestinal epithelial cells (86), the intestinal flora has the potential to facilitate CRC metastasis through modulating endogenous H_2_S synthesis and related pathways.

#### 2.2.2 Regulating mRNA methylation

The presence of the microbiota has been shown to induce epigenetic changes in mouse tissues at transcriptional level (87, 88). N6-methyladenosine (m6A), one of the epigenetic modification mechanisms of mRNA, could influence various fundamental biological processes (89). METTL3, the main m6A methyltransferase, is involved in the progression of several types of cancers, including acute myeloid leukemia (90), hepatocellular carcinoma (91), and lung cancer (92). In CRC, F. nucleatum has been shown to inhibit the Hippo pathway and activate the YAP signaling, leading to the suppression of METTL3 expression through the transcription factor FOXD3. The inhibition of METTL3 resulted in decreased m6A methylation of KIF26B mRNA, a gene associated with cell-cell adhesion and important for cancer cell invasion. Consequently, the expression of KIF26B were promoted, leading to enhanced tumor cell invasiveness. Therefore, F. nucleatum could induce epigenetic modifications in the KIF26B gene at transcriptional level through the YAP/FOXD3/METTL3 axis, ultimately facilitating the invasiveness of cancer cells (93).

**Figure 1:**
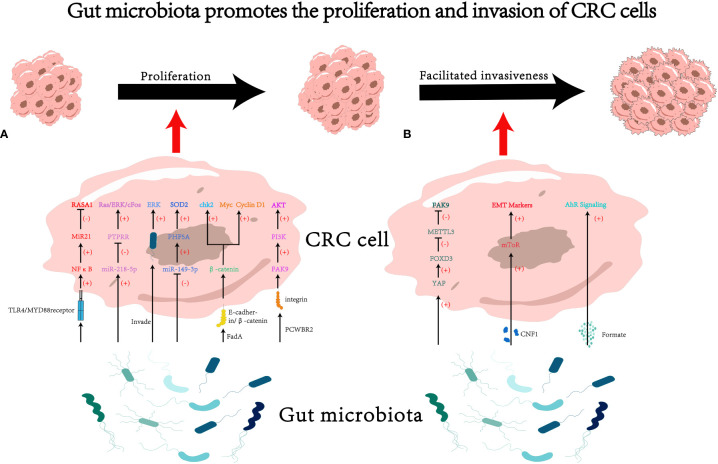
The impact of gut microbiota on proliferation and invasion of colorectal cancer (CRC) cells. **(A)** Gut microbiota may influence cancer cell proliferation through various pathways, such as modulating RNA-mediated targeting effects (33, 42, 46) and activating the cascades of cancer signaling (18, 50, 60, 64). **(B)** Gut microbiota may influence cancer cell invasiveness through various pathways, such as secreting microbiota-derived functional substances (70, 79), and regulating mRNA methylation (93). PCWBR2, putative cell wall binding repeat 2. CNF1, cytotoxic necrotizing factor 1.

In conclusion, gut microbiota is capable of promoting proliferation and invasiveness of CRC cells via multiple mechanisms (see **Figure 1** for details).

## 3 Gut microbiome promotes dissemination and survival of cancer cells

Most kinds of cancers rely on blood vessels, lymphatic vessels, and other channels for metastasis. Survival pressure like anoikis, shear forces, and immune attacks are exerted on cancer cells once they enter the circulatory system (68). Therefore, dissemination and survival are crucial prerequisites for cancer cells to complete metastasis. In recent years, studies have found that gut microbiota not only promotes cancer cell proliferation and increases their invasiveness but also facilitates cancer cell dissemination and survival (94–96). The mechanisms behind this include regulating intravasation of cancer cells to facilitate dissemination, participating in immune evasion to promote cancer cell survival, and modulating the tumor microenvironment to facilitate the formation of metastatic lesions.

### 3.1 Regulating intravasation to foster the dissemination of CRC cells

Structural and functional disruptions of vascular basement membrane (97), as well as tumor cell reprogramming (98), are two important processes involved in hematogenous metastasis of tumors. The former provides a physical basis for cancer cells to breach blood vessels and enter the bloodstream, while the latter enhances the intravasation and migration capabilities of tumor cells.

Under a high-fat diet, elevated levels of deoxycholic acid (DCA) in the host gut was detected, which enhanced vasculogenic mimicry in tumor tissues (99) —— the formation of structures that lack endothelial cells but possess normal vascular functions (100). This study suggests that the gut microbiota’s regulation of host metabolism may contribute to vasculogenic mimicry and promote tumor metastasis. However, the direct association between bile salt-hydrolyzing bacteria and intestinal DCA levels requires further investigation. Therefore, the mechanisms by which the gut microbiota regulates tumor vasculogenic mimicry through DCA still need to be further validated.

The adhesion of circulating tumor cells to endothelial cells and extravasation into pre-metastatic sites is an important process in tumor metastasis (101). Intercellular adhesion molecule 1 (ICAM1), a member of the immunoglobulin superfamily, has been shown to promote tumor cell adhesion to endothelial cells and facilitate metastasis (102). Its expression levels also positively correlate with tumor progression and metastasis in clinical settings (103). F. nucleatum could activate the NF-κB pathway by acting on the pattern recognition receptor ALPK1 on cells, thereby upregulating ICAM1 expression and promoting CRC cell adhesion to endothelial cells (96), ultimately facilitating metastasis of CRC.

### 3.2 Participating in immune evasion to promote the survival of CRC cells

Immune surveillance imposes strong selective pressure on cancer cells (104). The gut microbiota can directly or indirectly inhibit the function of immune cells, thus mediating immune evasion of tumor cells (61, 105–107).

The TIGIT (T cell immunoglobulin and ITIM domain) receptor is expressed on all NK cells and some other types of lymphocytes (108). F. nucleatum can directly interact with the TIGIT receptor through its surface virulence protein Fap2, thereby inhibiting the cytotoxicity of NK cells against cancer cells and ultimately inducing immune evasion of tumor cells (105). In addition to affecting the host’s innate immunity, the gut microbiota also regulates host adaptive immunity. Research by Jiang et al. has shown that succinate produced by F. nucleatum could inhibit the cGAS-IFNβ pathway, leading to reduced levels of chemokines CCL5 and CXCL10 in the tumor, thereby limiting the migration of CD8+ T cells to TME and suppressing the anti-tumor response of CD8+ T cells (109).

Myeloid-derived suppressor cells (MDSCs) from the bone marrow exert immunosuppressive effects through the depletion of amino acids and the expression of TGFβ and PD-L1 (110). Certain specific pathogens such as F. nucleatum and P. anaerobius can induce tumor-derived chemokine CXCL1 to recruit the MDSCs, thereby suppressing anti-tumor immunity (61, 107). The gut microbiota can also activate the TLR-calcineurin-NFAT-IL-6 signaling cascade on MDSCs, leading to the STAT3-dependent induction of the inhibitory protein B7H3/4, resulting in functional inhibition of cytotoxic T cells and ultimately promoting tumor immune evasion (111).

It’s worth noting that the effects imposed on the anti-tumor immunity by gut microbiota is a double-edged sword. A consortium of 11 bacterial strains was found to induce a strong CD8+ T cell response that boosted the efficacy of immune checkpoint blockade in mice (112). Other species such as Enterococus hirae could facilitate anti-tumor immunity in mice by enhancing CD8+ T cell anti-tumor responses when used in combination with cyclophosphamide chemotherapy (113). Bachem et al. discovered that butyrate, a microbiota-derived short-chain fatty acid (SCFA), enhances CD8+ T cell metabolism and promotes their differentiation into memory T cells (114). Similarly, microbiome-derived inosine could facilitate the differentiation of T_H_1 cells in an adenosine 2A receptor-dependent manner and consequently improve the antitumor effect induced by the ICB therapy (115). Since the adenosine 2A receptor has been demonstrated to inhibit T_H_1 differentiation *in vitro* as well as antitumor immunity *in vivo* (116–119) and only a few has reported that adenosine 2A receptor signaling can sustain T_H_1 and antitumor immunity (120, 121), the crosstalk between microbiota-derived metabolites, adenosine 2A receptor signaling and host immunity needs to be further investigated. In terms of clinical practice, a phase I clinical trial enrolling 20 patients have shown that fecal microbiota transportation (FMT) in combination with anti PD-1therapy could lead to a promoted immune status in patients with melanoma (122). These researches indicate that the correlation between gut microbiota and host immunity could be far more complicated and worth further investigation.

Besides regulating the anti-tumor immunity, the gut microbiota plays an important role in the development and maturation of the host immune function. Germ-free mice are unable to develop mature isolated lymphoid follicles (123). Additionally, the gut microbiota can regulate the function of different types of immune cells such as Treg cells, DC cells, and T cells, thereby establishing a normal intestinal immune homeostasis during early host development by balancing local pro-inflammatory and anti-inflammatory responses (124–126). Similarly, changes in the functional status of the immune system can change the composition of the gut microbiota. Activation of the AhR pathway in Th17/Th22 cells can induce the production of IL-22 and IL-17, which in turn can stimulate intestinal epithelial cells to secrete antimicrobial peptides, ultimately limiting the proliferation of pathogenic microbial communities (125). Individuals with immune deficiencies are more prone to dysbiosis of the gut microbiota, leading to various chronic inflammations (127).

These facts indicate that the gut microbiota-immunity axis is a complex bidirectional process. During the occurrence and development of tumors, changes in the gut microbiota are accompanied by immune dysregulation. The aforementioned studies have revealed various mechanisms by which the gut microbiota participates in immune evasion, providing a new perspective for a deeper understanding of the correlation between gut microbiota and the host immunity.

### 3.3 Modulating TME to facilitate colonization of CRC cells

TME consists of various cell components (128, 129), and its complexity has made it a tendency to view the TME as an organ itself (128). In certain situations, these components can produce bioactive factors and release them into the TME, thereby promoting tumor angiogenesis, invasion, and metastasis (130–133). Recent studies have found that there could be multiple kinds of bacteria with regulation effect in the TME besides the cell component. For instance, Xu and colleagues found that F. nucleatum could facilitate tumor metastasis in a CCL20-dependent manner (94). Although the only known receptor for CCL20—CCR6 is mainly expressed in immature dendritic cells, innate lymphoid cells, regulatory CD4 T cells, Th17 cells and B cells (134), a positive correlation between F. nucleatum-induced CCL20 expression and F4/80+ CCR6+ macrophage in lung metastasis tissues was observed (94). And they also found that F. nucleatum could directly promote the polarization of M2 macrophages in tumor tissues (94). Current researches have shown that M2 macrophages play important roles in immune suppression, tumor angiogenesis, and EMT (135–137). There are also studies showing that several bacteria such as segmented filamentous bacterium (SFB; Candidatus Savagella), ETBF, Bifidobacterium spp., F. nucleatum could modify the polarization of CD4^+^T cells into T_H_17 cells (138–141). T_H_17 cells has been indicated to foster an inflamed and tolerogenic TME (142, 143), providing a potential mechanism by which gut microbiota facilitates the future process of metastasis.

Not only is the microenvironment of the primary tumor important for tumor metastasis, but also the remodeling of the microenvironment in the metastatic foci plays a crucial role in the process of tumor metastasis. According to the “seed and soil” hypothesis, certain tumor cells can selectively settle in organs with suitable growth environments (144). Since the microenvironments of different organs vary, a particular type of tumor cells tends to preferentially colonize a specific organ (145). This preference may originate from the selective remodeling of the target organ by the primary tumor before metastasis occurs (146). Proteus mirabilis (P. mirabilis) and Bacteroides vulgatus (B. vulgatus) can modulate the hepatic immune niche by regulating the proliferation of Kupffer cells and inhibiting their phagocytic ability, ultimately fostering liver metastasis of CRC (19).

**Figure 2:**
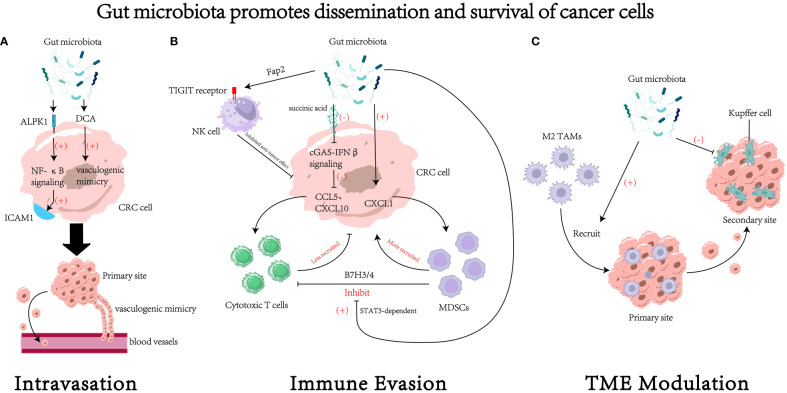
The impact of gut microbiota on the dissemination and survival of colorectal cancer (CRC) cell. **(A)** Gut microbiota could regulate the process of intravasation through various mechanisms, such as increasing the expression of intercellular adhesion molecule 1 (96) and promoting the formation of vasculogenic mimicry (99). **(B)** Gut microbiota could regulate survival of cancer cells by modulating tumor immune evasion through various pathways. For example, gut microbiota can inhibit the cytotoxic activity of natural killer (NK) cells through receptor-ligand interactions (105), reduce the recruitment of cytotoxic T cells (109), increase the recruitment of myeloid-derived suppressor cells (MDSCs) (61, 107), and foster the suppressive effects of MDSCs on cytotoxic T cells (111). **(C)** Gut microbiota may remodel the tumor microenvironment (TME) to facilitate the process of dissemination and colonization. For instance, it could recruit M2 tumor associated macrophages (M2 TAMs) to the primary site (94) and inhibit the function of Kupffer cells in the secondary site (19). DCA, deoxycholic acid. ICAM1, intercellular adhesion molecule 1. MDSCs, myeloid-derived suppressor cells. TAMs, tumor associated macrophages.

In conclusion, gut microbiota can facilitate the dissemination and survival of CRC cells via different ways(see **Figure 2** for details).

## 4 Conclusion

Studies on the correlation between gut microbiota and CRC can be traced back to 1951. Subsequent advancements in techniques such as 16S rRNA sequencing and metagenomic sequencing have made it possible to identify gut microbiota that are significantly associated with CRC. Multiple mechanisms have been proposed regarding how the gut microbiota regulates anti-tumor effects and participates in pathological processes especially metastasis of CRC in the following years (see **Table 1** for details). Consequently, CRC is a suitable model disease to investigate novel strategies for early cancer detection. Stool-based screening such as 16s rRNA sequencing is considered as a promising, non-invasive approach compared with colonoscopies (155). For bacteria widely participated in the initiation and progression of CRC, high-specific therapy strategies such as targeted antibiotic (156) and bioinorganic hybrid bacteriophage (157) has presented an attractive prospect for prevention and curation.

**Table 1:** Overview of milestones unveiling the correlation between gut microbiota and CRC.

Significance	Studies	Year
➢ Exploring the association between gut microbiota and CRC for the first time.	• Enterococcal endocarditis associated with carcinoma of the sigmoid; report of a case (147).	1951
➢ Identification and characterization of gut microbiota associated with CRC using RNA sequencing technology.	• Genomic analysis identifies association of Fusobacterium with colorectal carcinoma (148).	2012
➢ Elucidation of the mechanisms by which gut microbiota promote the occurrence and development of CRC for the first time.	• Fusobacterium nucleatum promotes colorectal carcinogenesis by modulating E-cadherin/β-catenin signaling via its FadA adhesin (18).	2013
➢ The association between gut microbiota and radiotherapy as well as chemotherapy.	• Enterococcus hirae and Barnesiella intestinihominis facilitate cyclophosphamide-induced therapeutic immunomodulatory effects (79).	2016
	• Gut microbiota modulates dendritic cell antigen presentation and radiotherapy induced antitumor immune response (149).	2020
➢ Cross-cohort studies of gut microbiota and its association with colorectal cancer using metagenomic sequencing technology.	• Meta-analysis of fecal metagenomes reveals global microbial signatures that are specific for colorectal cancer (150).	2019
	• Metagenomic analysis of colorectal cancer datasets identifies cross-cohort microbial diagnostic signatures and a link with choline degradation (151).	2019
➢ Gut microbiota and tumor immunotherapy.	• Anticancer immunotherapy by CTLA-4 blockade relies on the gut microbiota (152).	2015
	• Fecal microbiota transplant overcomes resistance to anti-PD-1 therapy in melanoma patients (153).	2021
	• Dietary fiber and probiotic s influence the gut microbiome and melanoma immunotherapy response (154).	2021

CRC, colorectal cancer; PD-1, Programmed cell death protein-1; CTLA-4, cytotoxic T lymphocyte-associated antigen-4.

Recent studies have implicated that oncogenesis and progression of cancers could be consequences of the dysregulated immunologic function. Mechanisms like immune checkpoint shed a light on the complicated networks between cancer and immunity. Still, such theories cannot fully illuminate the role of systematic factors, such as exercise, diet and aging, in crosstalk between cancer and immunity. As one of the earliest encountered environmental antigens in the human body, gut microbiota could facilitate cancer metastasis and modulate immune response through mechanisms mentioned afore, which may explain the role of systematic factors in cancer and immunologic function. Nonetheless, more efforts should be dedicated to further unveil the mechanisms by which systematic factors such as gut microbiota regulate the process of cancer oncogenesis and progression.

## Author contributions

SY: Writing – original draft. SW: Writing – review & editing. BX: Writing – review & editing. CP: Writing – review & editing.
